# Creating together, growing together: a qualitative study of adolescent experiences in an inclusive, co-creative performing arts project

**DOI:** 10.1080/17482631.2025.2582287

**Published:** 2025-11-11

**Authors:** Eva Mari Andreasen, Thomas Westergren

**Affiliations:** a Kilden Performing Arts Centre and Faculty of Fine Arts, University of Agder, Kristiansand, Norway; b Department of Public Health, University of Stavanger, Kristiansand, Norway

**Keywords:** Inclusive arts, co-creative performing arts, adolescence, young adults, qualitative study, well-being

## Abstract

**Purpose:**

Adolescence is a formative period marked by exploration, identity formation, and a growing need for belonging. Co-creative performing arts practices may support learning and relationship-building across diverse groups of young people. This study explored how adolescents without disabilities, enrolled in mainstream educational programmes, experienced and reflected on their participation in a long-term, inclusive, co-creative performing arts project (*SPOR*).

**Materials and methods:**

A qualitative inductive design with three focus groups was used, grounded in phenomenological, hermeneutic, and existential traditions. A meaning-oriented approach rooted in reflective lifeworld research ensured methodological coherence, and reflexive thematic analysis facilitated a systematic yet adaptable exploration of participants’ lived experience.

**Results:**

The analysis yielded two themes: *Finding pride in the challenge of co-creation* and *Tracing essentials for living*. The latter comprised three interrelated subthemes: *Seeing oneself and society with new eyes*, *Unlearning stereotypes through sustained encounters*, and *Grieving the heartfelt community*.

**Conclusion:**

Participants described emotional growth, mutual recognition, and a sense of belonging and mattering arising from collaborative artistic engagement with peers with disabilities. The findings suggest that inclusive co-creative performing arts can promote adolescents’ psychosocial development and well-being, while highlighting the need for supportive structures that sustain and extend such experiences into everyday life.

## Introduction

### Adolescent well-being in a changing society

Adolescence and early adulthood are formative and sensitive phases of life marked by biological, psychological, and social changes. During this time, young people develop their identities, seek belonging and navigate shifting social expectations. Environments that provide stability, recognition and relational support can strengthen young people’s ability to cope with challenges and maintain well-being, whereas adverse experiences may have lasting effects on identity formation, mental health and social participation (Marquez et al., 2023; Mesman et al., 2021; Uhlhaas et al., 2023).

Globally, mental health conditions among young people are on the rise. These include anxiety, depression and suicidal ideation (NOVA, 2024; WHO, 2023). Studies highlight the particular vulnerability of adolescents to social stressors, such as academic pressure, traditional gender expectations and social media exposure (Keyes & Platt, 2024). These challenges may compromise well-being and contribute to negative outcomes such as poor body image and emotional stress (Merino et al., 2024; Shannon et al., 2022).

At the same time, sociopolitical developments across the world, including Norway, have contributed to a more exclusionary climate (CIRCLE, 2025; van der Brug et al., 2025). In the United States, this has coincided with growing resistance to diversity and inclusion in education, including a backlash against curricula addressing race, gender and social justice (McGowan et al., 2025). Such developments risk deepening the exclusion of already marginalised groups, including youth with disabilities (Lindsay et al., 2015, Lindsay et al., 2023). These trends highlight the urgent need for inclusive spaces to foster a sense of belonging, diversity, and meaningful participation through creative engagement.

### The potential of inclusive co-creative arts

One response to these challenges lies in inclusive, co-creative performing arts. Norwegian cultural policy (Ministry of Culture, 2021) encourages the development of cultural arenas for, with and by young people, supporting active participation rather than passive consumption. Co-creative performing arts often draw on real-life experiences in which **“**ordinary” people (i.e., those who are not trained actors) are invited to engage in storytelling (Takács, 2022). Co-creative performing arts projects aim to expand ideas about what theater is and who it is for. It is a social process that allows for personal expression and supports relationship building to bridge differences (Cases-Cunillera et al., 2024). When these processes occur in inclusive contexts, they create spaces for different needs, voices and ways of participating (Gjærum & Rasmussen, 2010). In inclusive co-creative arts, people with and without disabilities create together and actively embrace diversity (Cardol et al., 2025). A key aim is often to challenge and dismantle ways of thinking and social systems that present being able-bodied as the ideal standard for being human (Cardol et al., 2025).

### Existing research on participatory arts and inclusion

An expanding body of research has highlighted the potential of participatory art to promote well-being, self-confidence and social connection. Daykin et al. (2021) found in a qualitative systematic review that participatory arts can foster belonging and connection by creating inclusive spaces where people feel recognized and safe. The review also highlights how such projects may build bridges across differences and support wider community engagement. While most research focuses on short-term initiatives, some longitudinal studies provide tentative insight into how arts participation may relate to adolescent development. For instance, Mansour et al. (2018) followed over 600 students aged 11 to 19 over one academic year and found reported associations between participation and students’ developing arts self-concept, which may have implications for engagement and motivation. A rapid review by Zarobe and Bungay (2017) suggests that structured group-based arts activities in community and extracurricular school settings may contribute to young people’s mental wellbeing. These activities support self-confidence, self-esteem, social relationships and a sense of belonging. The review also highlighted the role of arts in fostering identity development and emotional expression. Hatala and Bird‐Naytowhow (2020) describe how theater-based storytelling and performance among Indigenous youth in Canada provided a safe and culturally rooted space for exploring identity, belonging, and wellness through creative self-expression and cultural reconnection.

Many extant studies have focused on how inclusive performing arts benefit people with disabilities. For example, Grabowski et al. (2024) analysed interviews with youth with intellectual disabilities participating in an inclusive theater program, while Saar et al. (2025) combined ethnographic observations with participants’ reflections from a dance project. These studies underscore the transformative potential of such artistic involvement. However, much less is known about the experiences of adolescents without disabilities who take part in inclusive, co-creative art projects. Nevertheless, such participation has great potential for development. Inclusive and co-creative projects generally provide valuable opportunities for adolescents with and without disabilities to develop positive attitudes, social and emotional skills and leadership qualities (Clement & Freeman, 2023; Edwards et al., 2021; Jacob et al., 2024). These experiences can help build a more inclusive and empathetic society, benefiting all participants involved (Clement & Freeman, 2023; Schuh et al., 2015). In theater contexts, adolescents without disabilities have reported experiencing increased empathy and understanding when working with peers with disabilities (Nijkamp & Cardol, 2020). However, more research is needed to deepen our understanding of such experiences and their implications. In particular, further research is needed to understand the personal and social meanings of such engagement and to inform the development of future practices (Grabowski et al., 2024; Nijkamp & Cardol, 2020).

### Theoretical framework

This study was informed by the interrelated concepts of *belonging*, *well-being* and *mattering. Belonging* refers to a sense of connection with people, places and communities (Allen et al., 2021). It is influenced by individual capacities, opportunities for social interaction, motivation to participate and the perceptions of being included. As a basic human need, belonging is closely linked to well-being, resilience and mental health (Allen et al., 2021). *Well-being* is a multidimensional phenomenon involving emotional, psychological and relational dimensions. Following Ryff’s (1989) model, we view well-being as self-acceptance, positive relationships, autonomy, environmental mastery, purpose in life and personal growth. *Mattering* is a psychological need essential for the physical and mental health of adolescents and young adults (Flett et al., 2019), encompassing the feeling of being valued by others and the perception that one’s contributions make a difference (Prilleltensky, 2020). Mattering is interconnected with wellness and fairness, and these psychological goods collectively promote well-being on individual, interpersonal and societal levels (Prilleltensky et al., 2023)*.* These concepts will guide the discussion and help interpret how adolescents made sense of their participation.

### The current study

This study aimed to explore how adolescents without disabilities, enrolled in mainstream educational programmes, experienced and reflected on their participation in *SPOR*, a long-term, inclusive and co-creative performing arts project. The following research questions guided the study:

1. How do adolescents experience participation in an inclusive, co-creative performing arts process, and what meanings do they assign to the process?
2. What potential impact might a co-creative performing arts process have beyond the project itself?

## Materials and methods

### Study context

The study took place within *SPOR*, an inclusive co-creative performing arts project developed and led by Kilden Performing Arts Centre, a regional performning arts centre in Southern Norway (Kilden, 2025), in collaboration with local upper secondary schools. The project has been carried out in several iterations since its inception, each involving a new group of participants and culminating in a public performance. The word *SPOR* means **“**mark” and/or **“**trace” in Norwegian, reflecting the project’s ambition to leave a lasting imprint on those involved. Over a period of seven months, students from both general and special education programmes collaborate to develop and present an original stage production. Each participant plays an active role in shaping the final production, which integrates film, text, dance, song, and physical expression. Drawing on applied arts (Bishop, 2023), which encompasses participatory and inclusive performance practices as described in the existing literature (Nicholson, 2022; Seip, 2022; Takács, 2022), this process enables participants to explore and communicate both personal and shared narratives through artistic forms such as storytelling, music and movement. Students work alongside professional artists, educators and peers with disabilities, and are supported by extensive artistic and technical resources. Adaptive strategies, including flexible rehearsals and on-stage modifications, are used to ensure accessibility. The final performance is staged publicly at the performing arts center and features orchestral music composed specifically for the project and performed live by a professional symphony orchestra. The participants are usually 17–18 years old when the project starts, and although some may have turned 19 by the time of data collection, they are referred to as adolescents in this article, in line with developmental psychology (Zarrett & Eccles, 2006).

Although the first author is affiliated with the performing arts centre, neither of the researchers was involved in the artistic or educational activities of the project. Both researchers have professional backgrounds as registered nurses with experience from paediatric hospital departments and are experienced in qualitative methods. They also bring personal insights into the research as parents to children and adolescents in the age range between 9 and 23 years.

### Design

This study employed a qualitative inductive design, based on three focus groups. The research design was inspired by **“**the third way” described by Dahlberg and Dahlberg (2020), who highlight the inseparability of subjectivity and objectivity, as well as the interrelation of description and interpretation. We conducted thematic reflective analysis according to Braun and Clarke (2022), also guided by the principles of openness, bridling and flexibility, as described by Lindberg et al. (2024), for thematically analysing and describing meaning-oriented subjective experiences.

The focus groups were well suited for adolescents, as the group context tends to create a more relaxed and supportive atmosphere than individual interviews (Daley, 2013). Furthermore, focus groups enable participants to reflect collectively, build on each other’s thoughts, and engage in both agreement and disagreement (Parker & Tritter, 2006). Such interactions can generate rich, nuanced data by revealing how understanding develops through dialogue (Adler et al., 2019). Moreover, the format allows for more participants to be included than with individual interviews would permit, while offering the psychological safety of being in a group setting (Daley, 2013). These qualities made focus groups a good fit for exploring how young people made sense of, and assigned meaning to, their experiences through their involvement in an inclusive, co-creative art project.

In line with the principles of collaborative and inclusive research (Beresford, 2013), elements of co-production were embedded in the planning and design of the study at both the consultative and participatory levels. During the planning phase, key staff at the performing arts centre, including *SPOR*’s artistic director, contributed to both the study design and the development of the interview guide. Their experience with inclusive artistic processes informed the research about the realities of the project and contributed to the interview questions that reflected their artistic language and the context of the project. While the director was not involved in conducting or analysing the data, their input helped strengthen the relevance and clarity of the interview themes. This approach reflects a pragmatic form of co-production that prioritises dialogue with field actors to enhance the quality and relevance of research without compromising the independence of the analysis (Hackett, 2005).

### Participants

The inclusion criterion was that participants had to be students from mainstream educational tracks at one of the upper secondary partner schools who took part in the *SPOR* project and were actively involved in the co-creative process. At the time of data collection, the participants were aged 18–19 and enrolled in General Studies programmes at an upper secondary school in Norway.

### Recruitment

Participants were recruited to participate in focus groups through the teacher of the elective Music, Dance and Drama course at one of the partner upper secondary schools, in close collaboration with coordinators of the *SPOR* project. All students who met the inclusion criteria were invited to participate, guided by the principles of information power (Malterud et al., 2016), which suggest that a sample’s adequacy depends not solely on size, but on the richness and relevance of the data. This approach also ensured that everyone eligible had the opportunity to participate. All invited participants received comprehensive written and verbal information about the study before providing informed consent. Of 31 eligible students, comprising 29 females and two males, 24 female students agreed to participate. The two male students who were eligible chose not to participate. While this represents a limitation, participation was voluntary, and no one was excluded. The gender imbalance reflects the general composition of the Music, Dance and Drama programme in this context, which tends to have a predominance of female students. The procedure was designed to minimise potential authority pressure from teachers, as participation was confirmed solely by voluntary attendance and could be withdrawn at any time without any consequence or teacher involvement.

### Data collection

Three focus groups, each comprising eight participants, were conducted by the authors two weeks after the final *SPOR* performance, in a classroom at one of the participating upper secondary schools. More students than anticipated volunteered for participation, and within the limited timeframe allocated by the school, we decided to increase the number of groups from two to three. Moreover, structuring the discussions within a shorter timeframe was considered appropriate as it may help young people to be fully concentrated on the topic without compromising reflexive richness (Krueger & Casey, 2009).

Prior to focus the groups, we held a joint 10-minute briefing session to prepare students and explicitly informed all participants that the aim was to explore their genuine experiences and that there were no right or wrong answers. Emphasis was placed on creating a safe and open environment, particularly given that the interviews were conducted at the same school where participants were regularly taught and assessed. This approach was intended to minimize the perceived pressure to provide socially desirable responses and encourage honest reflection. The dialogue was designed as an open, in-depth conversation to ensure that rich and relevant data would be generated, with participants encouraged to reflect as they spoke. The moderator and the co-moderator were seated opposite one another, enabling eye-contact with all participants to actively invite those less extroverted.

A semi-structured interview guide was used to support the dialogue. Examples of questions are as follows: *What was it like to take part in SPOR? How did you experience performing and presenting the production on stage? What did you learn from being involved, about yourself and about others? What marks has SPOR left on you, and what marks do you think will stay with you in the future?* The focus groups, each lasting 30 minutes, were conducted in April 2025, with students assigned to the groups by their teacher. The groups were conducted in Norwegian and audio-recorded, with the participants’ consent, using *Nettskjema*, a secure recording and AI transcription tool approved by the University of Stavanger (UiS). All 24 students participated actively, sharing and reflecting collaboratively with honesty and vulnerability in their experiences with *SPOR*. Despite the narrow and focused timeframe, the discussions resulted in a meaningful and thematically rich dialogue, aligning with Malterud et al.’s (2016) criterion of high information power. Each group confirmed and elaborated on experiences raised in the others, contributing to a reciprocal and cumulative understanding.

### Analysis

The first author reviewed and cross-checked the automated transcriptions to ensure fidelity to the participants’ original statements. The analysis followed Braun and Clarke’s (2022) six-phase reflexive thematic analysis, informed by a meaning-oriented lifeworld approach (Lindberg et al., 2024). This enabled an iterative and layered interpretation, shifting between semantic detail and deeper, at times implicit, meanings. The principles of openness and bridling were operationalised by holding back early interpretations and remaining attuned to the complexity of participants’ lived experiences.

The first author led the analytical process with active and reflexive engagement. The second author reviewed the initial codes, contributed to refining the thematic structure through discussions with the first author, and provided critical feedback on emerging drafts. During the first phase, we familiarised ourselves with the data by conducting interviews, writing post-interview reflections and rereading transcripts multiple times. During the second phase, we systematically coded the data using NVivo (version 15) and conducted two full rounds of coding. Initial coding was conducted inductively, staying close to participants’ own words to emphasise an experiential and lifeworld-oriented perspective. This is in line with Braun and Clarke’s (2022) emphasis on meaning-making and Lindberg et al.’s (2024) emphasis on lived experience and openness to existential meaning. Code labels were reviewed and refined, and related codes were merged where appropriate. This approach emphasises the researcher’s active role in developing themes through an iterative process of reading, coding and interpretation. For instance, the statement “I thought it would be like one of those preschool shows where nothing works” was initially coded as *skepticism about quality* to reflect the participant’s early skepticism about the process. Similarly, “we made it work” was coded as *co-creation through effort* to highlight how participants experienced co-creation as a joint effort with a successful outcome. These codes were developed inductively and were further revised to *initial doubt* and *collaborative achievement* (see Table I for illustration). Rather than seeking thematic saturation, the analysis was driven by curiosity, reflexivity and attention to both semantic and latent levels of meaning.

During the third phase, initial themes were developed by identifying patterns of shared meaning across codes. The aim was to move beyond surface-level topic summaries and to explore participants’ lived experiences. In the fourth phase, the themes were reviewed using a bridled approach to interpreting participants’ responses. This meant deliberately holding back from jumping to conclusions or imposing theoretical assumptions too early in the process. Instead, the authors revised the coded material with an open and reflexive stance, allowing the meaning to emerge gradually through re-reading and discussion. For example, when reviewing the theme that later became *unlearning stereotypes through encounter*, the authors initially considered labelling it as *new perspectives*. However, upon closer examination of participants’ language and interaction, the authors recognised that the process they described involved more than simply gaining new insights. During the fifth phase, the authors collaborated to refine and describe the final themes from a meaning-oriented perspective. For example, a theme named *pride and achievement* was rephrased as *finding pride in the challenge of co-creation* to reflect the dynamic process of growth and collective accomplishment, evident in the transcripts. Finally, during the sixth phase, the authors wrote the full Results section, which was reviewed and commented on by both authors to ensure coherence and critical depth. Table I illustrates how selected excerpts from the transcript were first coded and then grouped under a common theme as part of the thematic analysis.

### Ethical considerations

The study was conducted in accordance with the principles of the Declaration of Helsinki (World Medical Association, 2013). Approval concerning data privacy and confidentiality was granted by *SIKT*, the Norwegian Agency for Shared Services in Education and Research, reference number 270379, on behalf of the University of Stavanger (UiS). The legal basis for data processing was grounded in the public interest, in accordance with the General Data Protection Regulation (GDPR Article 6[1][e]) and the Norwegian Personal Data Act (§ 8). In line with Norwegian law, approval from a local institutional review board was not required for this type of research. The Faculty of Health Sciences at UiS is responsible for ensuring compliance with relevant ethical procedures and legislation, including the Declaration of Helsinki and national research ethics guidelines.

All participants received both written and oral information about the study. Informed consent was obtained orally from all participants, in line with the approved protocol and based on a recommendation from *SIKT*, which assessed this approach as ethically and legally appropriate under the legal basis of public interest (GDPR Article 6[1][e]). The *SPOR* project is a public initiative involving a diverse group of young participants with varying levels of reading and writing proficiency. To ensure accessibility and equal participation across the entire project, oral informed consent was used consistently for all participants, in accordance with the approved ethical protocol. The oral consent process was documented in writing by the teacher immediately following each consent conversation. Those who gave consent were scheduled, in collaboration with their teacher, to participate in one of three focus groups. Attendance served as a secondary confirmation of consent. Participants were free to withdraw at any time simply by choosing not to attend, without their teacher noticing or being informed. However, more participants than we expected volunteered and showed up, as also described above. In collaboration with schools and the performing arts centre, participants took part voluntarily and retained the right to withdraw at any stage, whether from the co-creative process, the public performance, or the subsequent research, without any negative consequences.

Although the discussions could evoke emotional vulnerability, they also offered participants a rare opportunity to reflect on meaningful experiences and contribute to research that aims to improve inclusive practices for others. This potential impact was considered to outweigh the minimal risk involved.

All personal data were anonymised, with particular attention paid to the risk of indirect identification in small educational settings. Given the first author’s affiliation with the performing arts centre, particular attention was paid to maintaining critical distance and analytical independence. The analysis was conducted off-site, and the findings were not shared with colleagues at the performing arts centre during the analytical process. The external academic perspective of the second author provided an important counterbalance in both data collection and analysis.

**Table I.** Example of transcript, codes, and generated theme.

Quote	Code	Theme
*“In the beginning, during those rehearsals, I often thought this wasn’t going to work. I thought it would just be like one of those preschool shows where nothing works, where nothing fits together.”*(Focus Group 1)	Initial doubt	Finding pride in the challenge of co-creation
*“It was fun to see how much control many of them had. [.] But then it actually worked pretty well, because we made it work.”*(Focus Group 1)	Collective achievement	
*“I’ve never stood on a stage before. It’s so much fun. A lot of adults, especially many adults, have said that it kind of gave them hope for us young people. That we can actually make something beautiful, for once.”*(Focus Group 3)	Fun, giving hope, make beautiful for once	

## Results

The participants’ experiences with the *SPOR* project were summarised in two overarching themes: (1) *Finding pride in the challenge of co-creation* and (2) *Tracing essentials for living*. The second theme comprised three interrelated subthemes: (2.1) *Seeing oneself and society with new eyes*, (2.2) *Unlearning stereotypes through sustained encounters,* and (2.3) *Grieving the heartfelt community*.

### Finding pride in the challenge of co-creation

This theme explores participants’ experiences of the artistic process of the co-creative performance project. It centres on the collective journey of creating something together, overcoming challenges and feeling pride in what was ultimately achieved. The project began with uncertainty and skepticism. Several of the young participants expressed doubts about whether the project would be successful and described the first joint rehearsals as confusing and lacking clear direction. The rehearsal periods were also experienced as being long and demanding. One participant said: “During the first rehearsals at school, I didn’t think it would turn out to be such a good performance. It was just little things here and there, and it took a really long time to rehearse” (Focus Group 3).

Gradually, a sense of ownership developed. The participants were given increasing responsibility in the production, and the structured work towards a stage performance, in close collaboration with a peer with disabilities, gave them a tangible goal to pursue. Being recognised as key contributors in a professional artistic process gave the young participants a sense of being important to each other, to their peers with disabilities, and to a broader community. The artistic framework created predictability and a shared purpose to collaboratively create meaningful performance. Through this, a sense of being part of something meaningful became evident, and participants expressed pride in being viewed as responsible and capable young people. Some reported a sense of mastery from learning lines, remembering choreography, and remaining focused, but all participants emphasised that the strongest sense of achievement came from supporting others, particularly their peers with disabilities, to ensure the group’s success as a whole. This experience allowed them to contribute something greater than themselves, generating a source of pride. One participant said: “At first it felt like their show, not ours. But as we got more involved, it became our performance too” (Focus Group 2).

Performing for an audience became a highlight. Participants shared how the performance had an emotional impact, with classmates, parents, and teachers visibly moved. As one participant shared: “People sent messages afterwards saying they were crying” (Focus Group 2). For most, the performance was a unique experience because they felt they were taken seriously as committed, thoughtful young people with something important to express. What they had achieved together, through practice, facilitation and close collaboration, stood out as something they were genuinely proud of.

“*I’ve never stood on a stage before. It’s so much fun. A lot of adults, especially many adults, have said that it kind of gave them hope for us young people, that we can actually make something beautiful for once.”* (Focus Group 3)

Simultaneously, the participants emphasised that the performance represented more than just the final act on stage. Many highlighted the process as more important than the final product. It was the journey towards the completed expression, with all its demands for effort and collaboration, that truly left a lasting impact. Several also noted that it had not always been easy. The process required patience, adaptation and persistence from both themselves and their partner with disabilities. However, the young participants pointed out that it was precisely through these challenges that a sense of community and pride appeared. One participant concluded: “We were all proud of what we made together” (Focus Group 3).

*“It was fun to see how much control many of them had. In the beginning, during those rehearsals, I often thought this wasn’t going to work. I thought it would just be like one of those preschool shows where nothing works, where nothing fits together. But then it actually worked pretty well because we made it work.”* (Focus Group 1)

It was about more than supporting students with disabilities or “helping out”; it was about being an active part of an artistic team effort. The performance became a symbol of what they had achieved together, a result of perseverance, learning and mutual trust.

#### Tracing essentials for living

This theme explores how participation in the *SPOR* project initiated processes that extended far beyond the stage. Through collaboration with peers with disabilities, participants gained new emotional and social insights. The experiences from the project continued to resonate with them, even after the project concluded. For many, what began as a co-creative process became a deeply personal journey. Several participants described tracing something essential to life. The process sparked a new way of understanding belonging and community. The adolescents shared stories of close encounters with students with disabilities. These encounters elicited new perspectives on themselves and others, as well as on the communities in which they wanted to participate.

In the analysis, these experiences are further examined through three subthemes: (1) *Seeing oneself and society with new eyes*, (2) *Unlearning stereotypes through sustained encounters*, (3) *Grieving the heartfelt community.*

#### Seeing oneself and society with new eyes

For many participants, being partnered long-term with a peer with disabilities was a new and demanding experience. They entered the project with uncertainty. Many did not know what to expect, were afraid of doing something wrong and were unsure how to communicate. Some described how they kept their distance at first, but gradually, over time and through shared experiences, friendships developed between these young people with different capacities and life experiences. Partnering with a student with disabilities meant learning to read body language, interpret signals and understand individual needs without a predetermined script. Many described this as challenging, but deeply educational. What initially seemed like a one-way responsibility developed into mutual relationships grounded in trust and recognition. The participants found that students with disabilities offered something equally valuable in return: openness, spontaneity and genuine presence. This shift in perspective prompted participants to reflect on the qualities they came to admire in their peers: “They are so honest. They’re not afraid to do what they want. That’s what I liked, it was fun and so real” (Focus Group 1). The relationships formed during the project enabled encounters across difference. Participants described the openness of students with disabilities as deeply inspiring*:* “They were just being themselves. It made me think about how much we all hold back in everyday life” (Focus Group 1).

Several participants described being deeply moved by the unconditional warmth and acceptance they were met with, which lowered the threshold for their own openness, allowing relationships to develop quickly. Many were surprised by how quickly strong friendships could form when trust and generosity were present. One participant reflected: “Even if you’ve just met, they’ll give you a hug. It teaches us that you can show love and share more of yourself” (Focus Group 2).

Participants often contrasted this with their everyday relationships with peers, in which there was less room for emotional openness. They experienced how students with disabilities made different kinds of emotional closeness possible: “We have more of an emotional connection with them than we do with each other. We tend to push feelings away” (Focus Group 3).

Being a source of steady support for someone else gave the participants a strong sense of meaning. Simultaneously, many gained a new understanding of interpersonal dynamics and what it means to be truly present in a relationship. This opened up space to reflect on their own worth and growing emotional awareness. Several expressed how they felt they were exploring something new, both in meeting someone different from themselves and in discovering new sides of themselves: “At the start, I didn’t know how to be around them. Now it just feels natural” (Focus Group 1).

*“It was really nice to show that side too. I’ve done different performances before, […] but this time, it wasn’t just about what I was doing. It was about being there for someone, taking care of someone and showing them what I was expressing. My parents noticed that this was really different. I felt it was a vulnerable side of me I hadn’t shown before.”* (Focus Group 2)

In the small moments of everyday collaboration, such as seeing one another, supporting each other and being present, participants discovered “a different map”, one that pointed towards a more open and relational way of being in the world. Walking alongside someone, rather than in front or behind, created space for reflection and for a kind of learning that could not have taken place in a traditional classroom: “I feel like they can teach us and show us that because they’re so open when we meet them, they show us that you can show love” (Focus Group 1). This experience of emotional openness often led participants to reflect on their own development and sense of self: “I don’t think I’m the same person afterwards. You change a little, but in a good way*”* (Focus Group 1).

*“I feel like it was very educational for us too. I’ve actually learned a lot from working with people with disabilities. I hadn’t done that before, so it was a valuable learning experience. You almost become more mature in your personality.”* (Focus Group 3)

In summary, this theme illustrates how being a partner in long-term collaboration led participants on the path of personal maturation. They discovered that they could be both a resource for others, and in need of support themselves, and that meeting others with openness and generosity changed who they were.

## Unlearning stereotypes through sustained encounters

Through the *SPOR* project, participants encountered experiences that challenged their preconceptions and initiated a process of critical reflection and the gradual unlearning of stereotypes. Many began the project with clear assumptions about who students with disabilities were, shaped by broader societal attitudes towards disability. They spoke about how society often places people with disabilities into rigid categories, but their reflections revealed that they had many of these same beliefs. It was only through close collaboration during rehearsals, shared responsibility, and collective effort that these biases were brought to the surface and disrupted. Several participants described their experience of getting to know a group after having been outsiders. Students who had once seemed distant or unfamiliar became individuals with personalities, a sense of humour and strengths. They were surprised to find out how capable, expressive and joyful their peers with disabilities were, far beyond what they expected. One participant reflected: “Some were just like us, just needed a bit more time. Others didn’t speak at all” (Focus Group 2).

During the discussion, the participants began to reflect on how they, consciously or unconsciously, helped maintain social divisions and expectations surrounding differences. It became clear that the process of unlearning was not only about learning something new about others but also about turning a critical gaze inward. Several began to recognise themselves as part of the structures they initially criticized. Through their experiences, they started to articulate that prejudice does not merely exist “out there”, but that they themselves were both carriers of and participants in these structures. One participant described this shift in powerful terms:

*“For example, he has a disability […], so people think he’s just like that, always joking and never serious. But that’s just rubbish! And it felt so good to show that. We got to show the audience something real. During the performance, it became so clear that there are so many thoughtful people here, and prejudice is just crushing. It destroys how they see themselves, and how others see them. And I think SPOR really helped us see that more clearly too, that there are so many wise people who just haven’t been given a chance.”* (Focus Group 1)

This reflection reveals how the participants came to see the damaging effects of stereotypical assumptions on interpersonal relations, self-perception and opportunities for participation. By co-creating the performance, they challenged these narratives in themselves and in the audience.

Several participants also expressed surprise at how much their peers with disabilities contributed to their final stage performance. The production became a landscape in which unexpected capacities and strengths were revealed. Many admitted that, initially, they had not believed their peers would be able to achieve such a high standard.

*“You don’t usually see a show where people with disabilities can pull it off just as well as the rest of us. I think people are a bit shocked that you can actually create something together with them because you do kind of put them in a box when you don’t get to know them.”* (Focus Group 3)

This recognition pointed towards a deeper awareness of how internalised assumptions, whether conscious or not, can obstruct genuine inclusion:

*“We’ve taught each other a lot. They’ve taught us a lot about how we meet others, even though we might be the ones who are supposedly more used to that—meeting people. But maybe we’ve actually learned more about how to meet people who aren’t like you, who are different.”* (Focus Group 2)

These insights prompted participants to begin exploring new ways of understanding closeness and community.

### Grieving the heartfelt community

When the project ended, several participants reported experiencing an unexpected sense of emptiness. What ended was not just a performance; it was a close-knit community that suddenly dissolved. As one participant noted, “It was a really hard transition” (Focus Group 1). Another said, “I am going to miss them; they’re people I have really come to care about” (Focus Group 3).

After weeks of daily rehearsals, shared tasks and intensive collaboration, there were no more gatherings or joint rehearsals, and for some, no further opportunities to meet. Many described it as a sudden rupture, like being in a bubble that suddenly burst. One participant expressed frustration: “You spend a whole year building relationships, and then it’s just cut off” (Focus Group 1). The sense of loss extended beyond individual relationships, touching on something larger: a deep, heartfelt sense of belonging and togetherness that they had not experienced previously. All the focus groups conveyed that this was something they felt was missing from their everyday lives. In *SPOR*, they experienced being part of something meaningful, being relied upon, and feeling that their presence mattered to the whole. When this disappeared, what remained was a heartfelt longing for what had been lost. As one participant reflected: “That’s the sad part […] during the intense last weeks when we were together all day, every day, and in vulnerable situations. They were nervous, excited, tired, sad and we were the ones who had to be there” (Focus Group 1). Another added: “When we talk about *SPOR* now, we smile, and it’s something positive, but I don’t feel like I carry that good energy with me when we’re not with them. Then we’re back to normal again” (Focus Group 2).

Some expressed frustration that there were no alternative spaces to continue what had started. Their questions were simple, yet powerful: Why must the relationship end? Why are there no more opportunities to be together like this? They pointed to a lack of structures or systems that could sustain what the project had initiated. For example, they mentioned the need for support-person programmes or low-threshold social arenas – not necessarily formal meetings, but simply spaces to meet again.

Simultaneously, the project led to tangible life choices. Some participants decided to become personal support workers because of their experiences, while others chose to pursue studies in special education. These decisions reflected the depth of the project’s impact on some participants, which touched them emotionally while also guiding their life directions.

However, the project’s end also brought tensions to light. Although the project aimed to promote inclusion, many felt that the relationships they had built were abruptly cut off, without any agency over how they ended. One participant said, “We were just told it’s over, that there would not be anything more” (Focus Group 1). This abrupt end felt jarring and difficult to grasp. Several expressed that it was strange for something powerful to stop. Some tried to maintain contact but found it difficult without the original shared context. As the participants reflected: “I do not know how I would even get in touch with them now” (Focus Group 1). The sudden absence of daily interactions was experienced as abrupt and emotionally challenging: “We had built strong bonds, and suddenly it was just over” (Focus Group 1). This sense of rupture was further emphasized by others who described the transition as jarring: “It was a hard transition. One week we saw them every day, the next not at all” (Focus Group 3).

This theme highlights how time-limited projects can lead to long-term needs. Being included in a community also sharpens awareness of its absence. Several participants said what they valued most was the realisation that there are places where they could be fully themselves and truly belong, yet they also expressed a clear wish that such communities would not appear and disappear, but be sustained in meaningful ways.

## Discussion

This study aimed to explore how adolescents without disabilities who are enrolled in mainstream educational programmes experienced and reflected on their participation in *SPOR*, a long-term, inclusive and co-creative performing arts project. The study examined how adolescents made sense of the collaborative process, what meanings they assigned to their involvement, and how the experience might impact them beyond the project itself. In the first theme, *Finding pride in the challenge of co-creation*, participants described a progression from uncertainty to a deep sense of pride in mastering complex collaborative processes. The performative framework enabled a tangible goal and collective responsibility, allowing participants to experience themselves as capable contributors to something meaningful. The second theme, *Tracing essentials for living*, captured how participants experienced emotional, ethical and social learning through the relationships they built. Across three interrelated subthemes, they described how encounters with peers with disabilities invited them to question social norms, unlearn stereotypes and reflect on the values and connections that matter most. These experiences awakened a longing for more heartfelt forms of community and belonging, connections that could extend beyond the project’s structured boundaries and into everyday life.

The results suggest that inclusive, co-creative art practices may support psychosocial development during adolescence, a time marked by emotional vulnerability, identity exploration and relational learning. Participants described development through sustained embodied collaboration, rather than abstract reflection. This co-creative process allowed them to view themselves as valuable contributors to meaningful collective efforts. These findings align with previous research suggesting that inclusive theatre can promote relational competence among adolescents (Nijkamp & Cardol, 2020). The co-creative project reflected both a performative collaboration and a relational community. The shared goal of creating together provided direction and meaning, while the work of rehearsing, adapting and caring for one another required emotional openness and mutual trust. In this way, psychosocial development was shaped through the interplay between artistic effort and human connection. Adding to this perspective, Williams et al. (2023) described how participatory arts programmes create emotionally safe and exploratory environments that resemble the mechanisms of change observed in therapeutic contexts. These programmes often disrupt everyday social hierarchies and open spaces for embodied play, shared vulnerability and reflective interaction, qualities that participants in the current project also identified as transformative. The results of this study add to a growing body of evidence suggesting that participatory arts can facilitate psychosocial development among those involved (Bungay & Vella-Burrows, 2013; Daykin et al., 2021; Williams et al., 2023; Zarobe & Bungay, 2017).

Participants described belonging as being emotionally present, seen and connected to others. They spoke of feeling close to their peers with disabilities, sometimes even closer than to their classmates they had known for years. These connections were marked by openness, generosity and mutual trust and shaped by their peers’ emotional expressiveness and spontaneity. This understanding of belonging aligns with recent theoretical developments that emphasise that belonging arises through the interaction of personal capacity, motivation, opportunities for connection and the perception of being included (Allen et al., 2021). In the project studied, belonging was supported by a clear structure, repeated interactions, and a shared goal that provided a framework for collaboration across differences. Belonging did not stem from shared group identity or similarity, but rather developed through emotional resonance and the experience of working together towards a common aim. This aligns with Easterbrook and Vignoles’s (2013) distinction between group-based belonging, which stems from shared social identity, and relational belonging, which is rooted in meaningful interpersonal connections. In this study, participants’ sense of belonging emerged not from being part of a predefined group, but from shared emotional experiences and collaborative efforts. Furthermore, Mahar et al. (2014) emphasise that a sustained sense of belonging depends on continuity, quality, and shared purpose. These factors were actively cultivated during the project.

Participants’ experiences point towards a multidimensional understanding of well-being consistent with Ryff’s (1989) model, comprising autonomy, self-acceptance, positive relationships, mastery, personal growth and purpose. They described their involvement as a path to greater confidence, deeper connections and awareness of themselves and others. They reflected on how they had grown both emotionally and socially. Participants described increased self-acceptance, particularly in how they understood their role within group dynamics and how they valued being part of something greater than themselves. These reflections support existing research on how inclusive arts initiatives can promote mental health, strengthen protective psychosocial factors and foster empathy (Cardol et al., 2025; Nijkamp & Cardol, 2020; Zarobe & Bungay, 2017). Notably, the young people in this study were not selected because they were viewed as vulnerable, nor did they describe themselves in these terms. Their experiences support a universalist view of health promotion in which everyone may benefit from participation in cultural life for all, not just those considered vulnerable. These insights reinforce Fancourt and Finn’s (2019) argument that engagement in the arts supports mental, social and emotional health across populations, and they parallel Rodriguez et al.’s (2024) observation that co-creative practices can foster belonging, meaning and psychosocial growth in diverse groups.

The participants’ experiences of mattering reflect Prilleltensky’s (2020) understanding of the concept as a dynamic balance between feeling valued and contributing value to others. In the project studied, the adolescents experienced both recognition and appreciation, and they had opportunities to make meaningful contributions, both to their peers with disabilities and to the audience. The project created conditions in which participants genuinely felt that their voices were heard and counted. Moreover, it actively challenged structural inequalities by ensuring that young people with and without disabilities could participate on equal terms, sharing responsibility and creative ownership throughout the process. This aligns with Prilleltensky et al.’s (2023) conception of mattering as a foundation for well-being and justice, built on reciprocity and recognition.

This study highlights how inclusive, co-creative performing arts can offer adolescents meaningful opportunities for psychosocial development, emotional growth and social learning. Through embodied art-based collaboration with peers with disabilities, participants discovered new ways of relating to others and to themselves. They described deepened self-awareness, emotional growth and a stronger sense of belonging and mattering. These findings contribute to the growing literature on participatory arts as a universal health-promoting practice, with potential to reduce stigma, foster inclusion and support adolescent development in a broader sense. Our study adds to the research field by highlighting how adolescents without disabilities interpret and are affected by inclusive collaboration. While many previous studies have explored the experiences of young people with disabilities (e.g., Grabowski et al., 2024; Saar et al., 2025), our study adds a complementary perspective by highlighting how adolescents without disabilities interpret and are affected by such collaborations. Experiences from the project indicate that co-creative performing arts can serve as a space for exploring values and ways of relating to others that are relevant beyond the artistic context. Participants reported a reshaping of identity and values, increased emotional openness, greater trust in others and a stronger sense of ethical responsibility. These forms of learning are relevant in schools, workplaces, leisure contexts and democratic participation. Several participants reflected on how they viewed others differently, while also considering their own role in maintaining or challenging social boundaries. These findings resonate with existing research showing that inclusive arts programmes can support identity development, facilitate social learning and reduce prejudice (Clement & Freeman, 2023; Hatala & Bird‐Naytowhow, 2020). For instance, in Hatala and Bird‐Naytowhow’s (2020) study, participants used theatre to try out new ways of being in the world, and some of these experiences were carried over into other parts of their lives. However, such transfers are context-dependent (Freebody et al., 2018). For this kind of learning to continue, adolescents need environments that recognise, support and build on the relationships and practices developed in inclusive, co-creative settings. This includes practical facilitation in schools and communities, as well as institutional cultures that value openness, diversity and mutual recognition.

At the same time, there is a risk that such projects, while well-intentioned, become isolated islands of ethical aspiration. If they are not followed up through sustained engagement or institutional support, the inclusive impulses they foster may fade, leaving behind only traces of good intentions. In this sense,inclusion risks becoming something that is performed rather than a transformation of relational practices. Moreover, while pride in the collective performance was a recurring theme, it is also worth asking how inclusive that pride truly was: did it extend to recognising the agency and artistry of peers with disabilities, or was it framed primarily through participants’ own emotional growth? These tensions underscore the need for a more critical examination of how inclusive arts projects can function simultaneously as as fragile beginnings that require long-term nurturing.

The study also highlights how fragile such experiences may appear from the perspectives of the adolescents when relational and structural foundations are no longer visible. Several participants experienced the end of the project as a loss, describing feelings of grief when the emotional bonds and collaborative ‘rhythm’ they had developed were no longer part of daily life. Our findings raise important ethical questions about time-limited projects: how to ensure that participation does not lead to disconnection or emotional rupture once the framework ends? To mitigate thsuch risksis, school health services and educational staff should be aware of the emotional needs that may arise after intensive collaborative experiences and be prepared to support adolescents in navigating these transitions. More broadly, the findings suggest that sustained and meaningful encounters with diversity during adolescence can play an important role in young people’s development. Inclusion, we suggest, cannot depend on isolated or time-bound efforts alone, but requires long-term interventions and policies that nurture inclusive and participatory environments (Anaby et al., 2013).

### Further research

To better understand the broader impact of inclusive arts, future studies should examine how participants transfer and apply learning from co-creative projects to other areas of life. This includes how experiences of diversity, dynamics of mutual mattering, shared belonging and dimensions of well-being shape social attitudes, relational patterns and ethical decision-making over time. It is also important to investigate how such projects affect participants with disabilities, as well as educational and artistic staff, and how mattering, belonging and well-being unfold from their perspectives. Future work should also explore how inclusive arts initiatives can be sustained across time and contexts, and what forms of structural and relational support are necessary to ensure lasting inclusion. Finally, comparative studies across roles, institutions and cultural contexts could provide important insights into the conditions that enable inclusive arts practices to support long-term flourishing, inclusion, mattering and relational well-being for all involved.

### Strengths and limitations

This study has several methodological strengths. The use of focus groups was well suited to the aim of exploring adolescents’ meaning-making in a shared, relational context. The group format enabled participants to reflect collectively, which contributed to the richness of the material within the limited time available. The combination of reflexive thematic analysis (Braun & Clarke, 2022) with inspiration from a lifeworld-oriented perspective (Lindberg et al., 2024) offered a flexible yet structured framework for exploring participants’ experiences. The study was grounded in inclusive and co-creative performing arts principles, with contextual input from practitioners contributing to the relevance and cultural sensitivity of the interview design. Ethical considerations were carefully addressed, with an emphasis on voluntary participation, emotional safety and researcher reflexivity. Importantly, the first author conducted the analysis away from the project site to maintain critical distance, and the second author’s external academic perspective served as a counterbalance throughout the process.

This study has some limitations. While the sample size was sufficient for the study’s aim, the participants were exclusively female, which may limit the transferability of the findings across gendered experiences. Furthermore, each focus group’s relatively short duration may have constrained the depth of individual reflections. Participants were also not involved in validating the interpretations. While this preserved analytical independence, it also limited opportunities for collaborative meaning-making at the final stage. Notwithstanding these limitations, this study offers a context-sensitive, methodologically robust contribution to understanding how inclusive, co-creative art practices can support adolescent well-being and development.

## Conclusion

This study demonstrates that inclusive, co-creative performing arts practices can promote adolescents’ psychosocial development and well-being. Collaborating with peers with disabilities gave the participants a sense of belonging and mattering. Participants’ reflections revealed a desire for more inclusive communities extending beyond the scope of this project, underscoring the importance of structures that can sustain and expand such relational experiences.

## Data Availability

Due to the sensitive and identifiable nature of the qualitative data and ethical approval conditions, the full transcripts are not publicly available. Anonymised excerpts supporting the findings of this study are available from the corresponding author upon reasonable request.
